# Early response to heat stress in Chinese tongue sole (*Cynoglossus semilaevis*): performance of different sexes, candidate genes and networks

**DOI:** 10.1186/s12864-020-07157-x

**Published:** 2020-10-27

**Authors:** Qian Wang, Xiancai Hao, Kaiqiang Liu, Bo Feng, Shuo Li, Zhihua Zhang, Lili Tang, Shahid Mahboob, Changwei Shao

**Affiliations:** 1 Key Lab of Sustainable Development of Marine Fisheries, Ministry of Agriculture, Yellow Sea Fisheries Research Institute, Chinese Academy of Fishery Sciences, Nanjing Road 106, Qingdao, 266071 China; 2 Laboratory for Marine Fisheries Science and Food Production Processes, Pilot National Laboratory for Marine Science and Technology (Qingdao), Qingdao, 266237 China; 3 College of Fisheries and Life Science, Shanghai Ocean University, Shanghai, 201306 China; 4 State Key Laboratory for Managing Biotic and Chemical Threats to the Quality and Safety of Agro-products, Ningbo University, Ningbo, 315211 China; 5 Department of Zoology, College of Science, King Saud University, Riyadh, 11451 Saudi Arabia

**Keywords:** Transcriptome analysis, Gonad, Heat stress, Heat shock protein, Cortisol, Epigenetic regulator

## Abstract

**Background:**

Temperature is known to affect living organisms and alter the expression of responsive genes, which affects a series of life processes, such as development, reproduction and metabolism. Several genes and gene families have been involved in high temperature responses, such as heat shock protein (*hsp*) family, Jumonji family and genes related to cortisol synthesis. Gonad is a vital organ related to the existence of a species. However, the comprehensive understanding of gonadal responses to environmental temperature is limited.

**Results:**

To explore the effects of environmental temperature on genes and gene networks in gonads, we performed acute heat treatment (48 h) on Chinese tongue sole (*Cynoglossus semilaevis*). Gonadal transcriptome analysis was conducted on females, pseudomales and males exposed to high (28 °C) and normal (22 °C) temperatures. A total of 1226.24 million clean reads were obtained from 18 libraries. Principal component analysis (PCA) and differentially expressed gene (DEG) analysis revealed different performance of sex responses to heat stress. There were 4565, 790 and 1117 specific genes altered their expression level in females, pseudomales and males, respectively. Of these, genes related to *hsp* gene family, cortisol synthesis and metabolism and epigenetic regulation were involved in early heat response. Furthermore, a total of 1048 DEGs were shared among females, pesudomales and males, which may represent the inherent difference between high and normal temperatures. Genes, such as *eef1akmt3*, *eef1akmt4*, *pnmt* and *hsp* family members, were found.

**Conclusions:**

Our results depicted for the first time the gonadal gene expression under acute high temperature treatment in Chinese tongue sole. The findings may provide a clue for understanding the responses of genes and networks to environmental temperature.

## Background

Water temperature is a major environmental factor that affects the development, metabolism and reproduction of aquatic ectotherms [1, 2]. Due to global warming, the water temperature of the ocean could rise between 1.1–6.4 °C by the end of the twenty-first century [3]. The increase of temperature may change the responsory genes expression, which can affect the regulatory networks. The response to high temperature has the difference between females and males [4]. The gonad is a vital organ related to the existence of a species. Therefore, the particular response mechanism in gonad under high temperature is needed to be clarified.

Many genes can respond rapidly to high temperature. For example, heat shock proteins (*hsps*) are molecular chaperones that function in protein folding, localization, secretion, and degradation [5]. Heat stress induces the expression of several *hsp* gene families. Of these, *hsp70* and *hsp90* are the most prominent ones. Up-regulated *hsps* have been observed in many fish species, such as red garra (*Garra rufa*), snakehead murrel (*Channa striatus*), and killifish (*Fundulus heteroclitus*) [6–8]. Cortisol is another factor in which production is increased by stress. Many studies on different fish species have found significantly higher cortisol concentration under high temperature and indicated that cortisol was involved in high temperature caused masculinization, such as pejerrey (*Odontesthes bonariensis*), olive flounder (*Paralichthys olivaceus*), and medaka (*Oryzias latipes*) [9–11]. Epigenetic regulators can link the external factors to internal gene regulation. Among them, the Jumonji family is one of the important factors. The members of this family, such as *jarid2* and *jmjd3* (*kdm6b*), can respond to temperature rapidly and affect sex-related gene expression [12, 13]. Although these genes and gene families have been involved in high temperature perception, a comprehensive understanding of gonad responses to environmental temperature is still lacking.

Recently, transcriptome analysis has become an excellent approach for investigating the responses of organisms to environmental changes, including heat [14–16]. Most of the reports are mainly focused on short-term high temperature stimulation in tissues, such as muscle and liver. In addition, there are several reports of the effects of long-term high temperature exposure on fish [17–19]. However, to the best of our knowledge, research is still lacking regarding the transcriptome data of fish gonads under short-term high temperature stimulation.

Chinese tongue sole (*Cynoglossus semilaevis*) is an economically important marine flatfish widely distributed in Chinese coastal waters, which possesses a ZW/ZZ sex-determination system. In our previous study, the genetic female: male ratio was close to 1:1 in this species. Approximately 14% of genetic females can spontaneously sex revered into phenotypic males (also known as pseudomales) [20, 21]. The genome of *C. semilaevis* has been well-sequenced, and the sex-specific simple sequence repeat (SSR) markers have been developed [21, 22]. *C. semilaevis* is an excellent model to evaluate the response to high temperature among different sexes. In this study, we conducted acute heat treatment on *C. semilaevis* and performed transcriptome sequencing on gonads of females, males and pseudomales. Our results may provide a clue for understanding gene and network responses to environment temperature.

## Results

### Gonadal transcriptome of *C. semilaevis*

To identify genes involved in response to heat stress in *C. semilaevis*, transcriptome sequencing was performed in the gonads of 3 females, 3 pseudomales, and 3 males of the control (CT_F1–3, CT_P1–3 and CT_M1–3; 22 °C treatment, CT group) and heat stress (HS_F1–3, HS_P1–3 and HS_M1–3; 28 °C treatment, HS group) groups. A total of 1354.19 million raw reads were generated from 18 libraries, which yielded 1226.24 million clean reads after quality control. An average of 88.87% of the clean reads was mapped to the high-quality *C. semilaevis* reference genome [National Center for Biotechnology Information (NCBI), Cse_v1.0, BioProject no. PRJNA73987] (Additional file 1). A total of 24,230 genes were detected in the transcriptome.

### Performance of sexes under heat stress

**Fig. 1:**
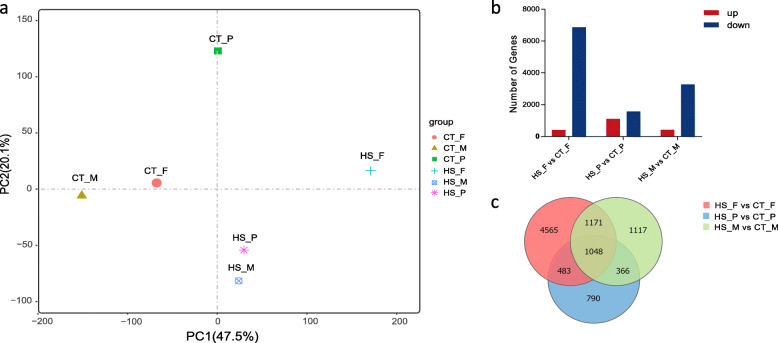
Analysis of differentially expressed genes (DEGs) under heat stress. **a** Principal component analysis (PCA) showed a clear cluster separation of the control (CT) and heat-stress (HS) groups. In CT group, females, pseudomales and males were apart from each other. In HS group, pseudomales and males formed one cluster while females exhibited another cluster. Each plot represents an average expression level of three biological replicants. **b** Number of DEGs identified from females, pseudomales and males between control (CT) and heat-stress (HS) groups. Red and blue colors indicate up-regulated and down-regulated genes in HS vs. CT, respectively. **c** Venn diagram depicting the distribution of the DEGs in HS vs. CT comparison in females, pseudomales and males

As shown in Fig. 1a, CT and HS groups were separated into two distinct clusters. Under normal temperature, females, pseudomales and males were apart from each other. After heat stress, females and males exhibited a separation, while pseudomales were close to males. The grouping was supported by differentially expressed gene (DEG) number analysis of three sexes between heat stress and normal temperature. The comparison of HS_F and CT_F showed 7267 DEGs (405 up-regulated and 6862 down-regulated genes). Comparison of HS_P and CT_P observed 2687 DEGs (1111 up-regulated and 1576 were down-regulated genes). In the comparison between HS_M and CT_M, a total of 3702 DEGs were identified (424 up-regulated and 3278 down-regulated genes) (Fig. 1b).

### Specific DEGs in each sex under heat stress

To detect different responses of sexes encountering heat stress, female, pseudomale and male-specific DEGs were analyzed. Females, pseudomales and males exhibited 4565, 790, and 1117 specific DEGs under heat stress (Fig. 1c).

**Fig. 2:**
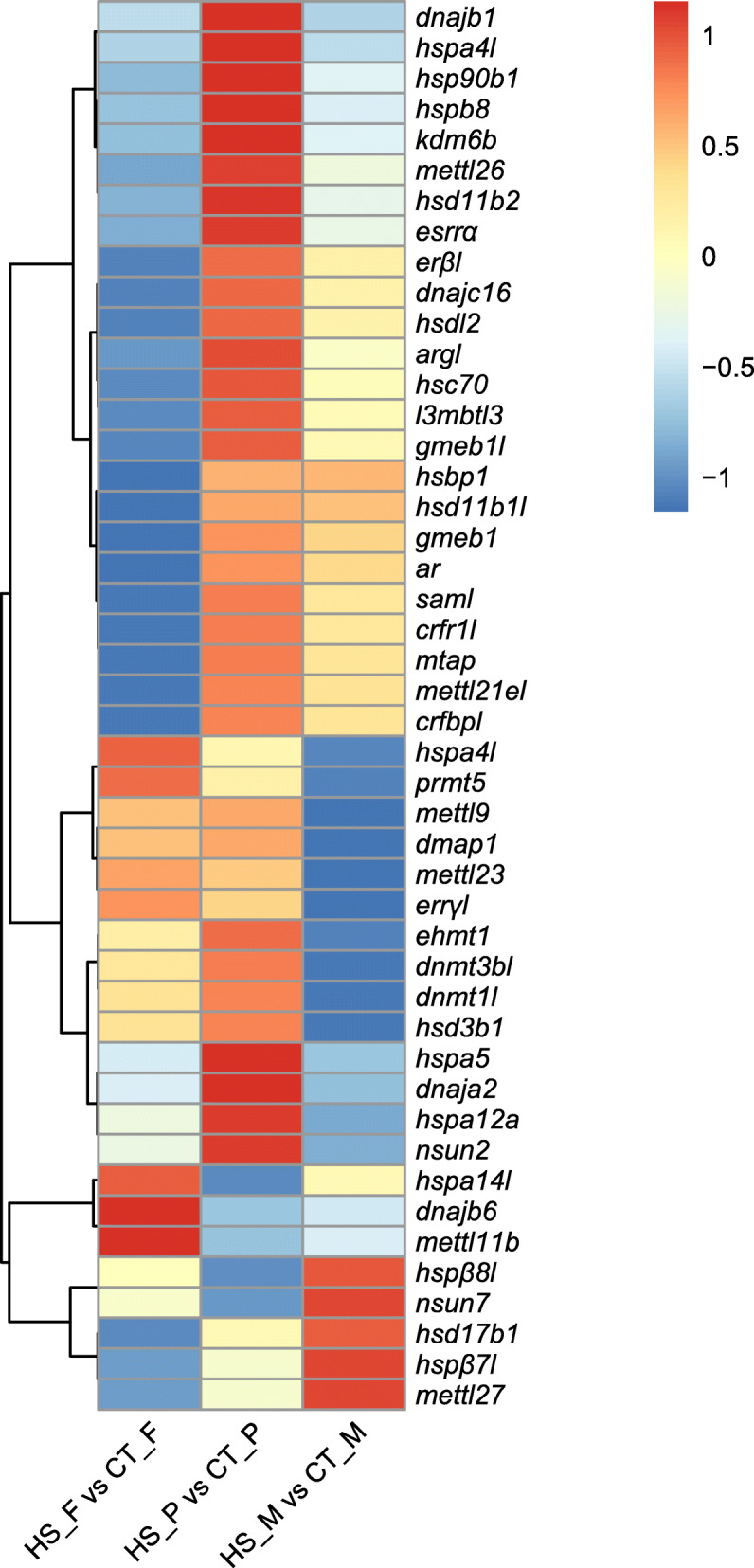
Heatmap analysis of sex-specific differentially expressed genes (DEGs) involved in high temperature response. Each row represents a gene listed on the right. Each column stands for a comparison of heat-stress (HS) vs. control (CT) groups in females, pseudomales and males. The expression of genes is color-coded from low (blue) to high (red) (DEGs, adjusted *p* < 0.001, log_2_FC > 1 or < − 1)

In this study, some members of DEGs related to the *hsp* gene family, epigenetic regulators, cortisol biosynthesis and sex steroid receptors showed sex-biased characteristics under high temperature. Heat shock protein family A (Hsp70) member 4 like (*hspa4l*), heat shock factor binding protein 1 (*hsbp1*) and heat shock 70 kDa protein 14-like (*hspa14l*) were differentially expressed in heat stress treated females. Heat shock protein family A (Hsp70) member 5 (*hspa5*), heat shock protein family A (Hsp70) member 12A (*hspa12a*) and heat shock cognate 70 kDa protein (*hsc70*) genes were found in pseudomales. DnaJ heat shock protein family (Hsp40) member A2 (*dnaja2*) and heat shock protein beta-7-like (*hspβ7l*) genes were found in males. Epigenetic regulation-related lysine demethylase 6B (*kdm6b*) was specifically down-regulated in females under heat stress. S-adenosylmethionine synthase-like (*saml*) was specifically up-regulated in pseudomales. DNA (cytosine-5)-methyltransferase 1-like (*dnmt1l*) was down-regulated in males. In addition, cortisol biosynthesis-related genes, including hydroxysteroid 11-beta dehydrogenase 2 (*hsd11b2*) and hydroxysteroid 17-beta dehydrogenase 1 (*hsd17b1*) were down-regulated in females under heat stress. On the other hand, estrogen related receptor alpha (*esrrα*) and androgen receptor (*ar*) were down-regulated in females, and estrogen-related receptor gamma (*errγl*) was down-regulated in males in HT vs. CT comparison (Fig. 2, Additional file 2).

### Gene ontology (GO) and Kyoto encyclopedia of genes and genomes (KEGG) analysis of sex-specific DEGs

There were 100, 55 and 85 GO terms significantly enriched in females, pseudomales and males, respectively (*p* < 0.05). In HS_F vs. CT_F comparison, the most enriched GO terms were immune system process, integral component of membrane, transferase activity-transferring glycosyl groups and positive regulation of actin filament polymerization. Besides, the GO term of regulation of histone H3-K4 methylation was significantly enriched (*p* < 0.05). In HS_P vs. CT_P comparison, the GO terms, including cell morphogenesis, RNA polymerase II transcription factor activity, actin binding and signal transducer activity, were mostly enriched. In HS_M vs. CT_M comparison, the most enriched GO terms were chromatin, nucleosome, tissue homeostasis and retina homeostasis (Additional file 3).

There were 103, 6 and 12 KEGG pathways significantly enriched in females, pseudomales and males, respectively (*p* < 0.05). In females, enriched KEGG pathways mainly referred to the immune system, such as NF-kappa B signaling pathway, cytokine-cytokine receptor interaction, natural killer cell mediated cytotoxicity and chemokine signaling pathway. In pseudomales, the most enriched KEGG pathways were cell adhesion molecules (CAMs), glycosaminoglycan biosynthesis-chondroitin sulfate/dermatan sulfate, amino sugar and nucleotide sugar metabolism and axon guidance. In males, the most enriched KEGG pathways included systemic lupus erythematosus, neuroactive ligand-receptor interaction, cellular senescence and pancreatic secretion (Additional file 3).

### Shared DEGs among three sexes under heat stress

**Fig. 3:**
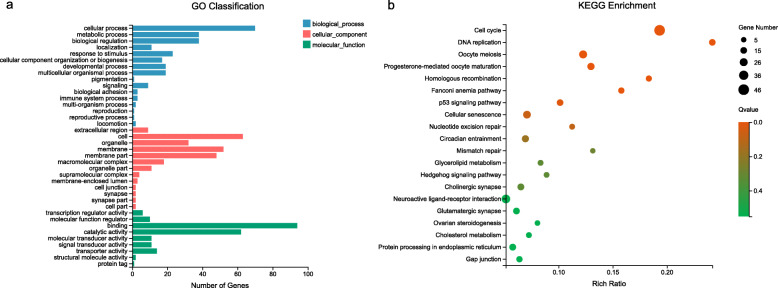
Analysis of differentially expressed genes (DEGs) shared among females, pseudomales and males. **a** Gene Ontology (GO) classifications of 1048 DEGs shared among three sexes in heat-stress (HS) vs. control (CT) groups. **b** Enriched KEGG pathways of 1048 DEGs shared among three sexes in heat-stress (HS) vs. control (CT) groups (*p* < 0.05)

The Venn diagram showed that there were 1048 shared DEGs among female, pesudomale and male individuals from the HS vs. CT comparison (Fig. 1c). EEF1A lysine methyltransferase 3 (*eef1akmt3*), EEF1A lysine methyltransferase 4 (*eef1akmt4*), phenylethanolamine N-methyltransferase (*pnmt*), and *hsp* family members, such as heat shock protein family B (small) member 1 (*hspb1*), heat shock protein family B (small) member 9 (*hspb9*), heat shock 70 kDa protein 1 (*hspa1*), heat shock transcription factor 4 (*hsf4*), and heat shock transcription factor 2 binding protein (*hsf2bp*) were found (Additional file 2). GO annotations of the shared DEGs were performed. In the biological process, cellular process, metabolic process and biological regulation were abundant. Cell, membrane and membrane part were abundant in the cellular component. In molecular function, binding, catalytic activity and transporter activity were enriched (Fig. 3a). A total of 26 KEGG pathways were significantly enriched (*p* < 0.05), including cell cycle, DNA replication and oocyte meiosis (Fig. 3b).

### Validation of RNA-seq by real-time qualitative polymerase chain reaction (qRT-PCR)

**Fig. 4:**
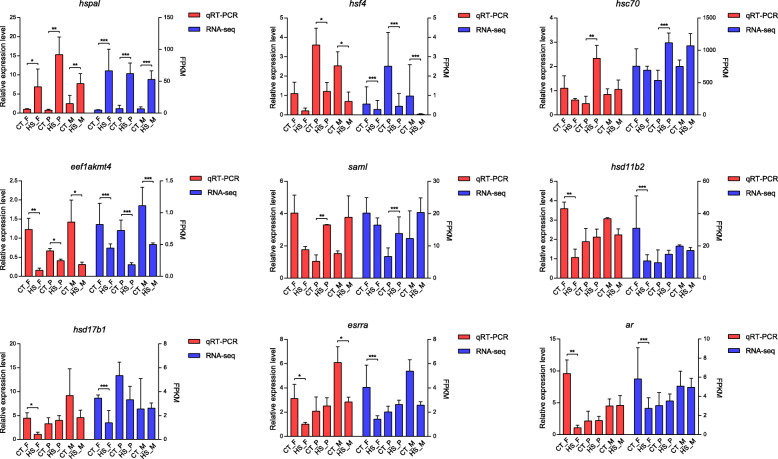
Quantitative reverse transcription-polymerase chain reaction (qRT-PCR) validation of RNA-Seq data. Nine DEGs, including *hspa1*, *hsf4*, *hsc70*, *eef1akmt4*, *hsd11b2*, *hsd17b1*, *saml*, *esrrα* and *ar* were selected for qRT-PCR. The left Y-axis represents the relative expression of DEGs, and the right Y-axis represents Fragments Per Kilobase Million (FPKM) determined by RNA-Seq. Data in females, pseudomales and males from each group (control group: CT_F, CT_P and CT_M; heat-stress group: HS_F, HS_P and HS_M) represent as mean ± S.E.M. of three biological replicates (*p < 0.05; **p < 0.01; ***p < 0.001)

To validate the transcriptome results, a total of nine significantly differentially expressed DEGs in at least one comparison group were selected for qRT-PCR analysis. Of these, three genes were the members of the *hsp* family, including *hspa1* (LOC103393458), *hsf4* and *hsc70* (LOC103395053). The *eef1akmt4* gene was a candidate regulator of HSP70. The *saml* gene (LOC103387328) was involved in S-adenosylmethionine synthesis and epigenetic regulation. The *hsd11b2* and *hsd17b1* genes were involved in cortisol synthesis. In addition, *esrrα* and *ar* genes were encoded for sex hormone receptors. For *hsf4*, RNA-Seq analysis showed significantly down-regulation in females, pseudomales and males under heat-stress, while qRT-PCR indicated no significant differences in females between high and control temperatures. For *esrrα*, RNA-Seq showed significantly down-regulation in females under heat-stress, while qRT-PCR indicated that there were significant differences in both females and males. Nevertheless, the down-regulation patterns of *hsf4* in females and *esrrα* in males were consistent between qRT-PCR and RNA-Seq. For the other seven genes, RNA-Seq and qRT-PCR showed similar significant up- and down-regulation patterns (Fig. 4). These results indicated that gene expression patterns determined by qRT-PCR were consistent with those determined by RNA-seq analysis, which supports the reliability of our transcriptome data.

## Discussion

Water temperature is an important external factor, which can significantly influence various physiological processes in fish. Females and males may have a sex-specific adaptation to the changing environments, which is a form of sexual dimorphism [2]. To access the gonadal performance in different sexes under heat stress, high-temperature stimulation in female, pseudomale and male *C. semilaevis* were performed in the present study, and the gonadal transcriptome data were analyzed.

In *C. semilaevis*, the response to acute high temperature was different among sexes. Females exhibited more DEGs than pseudomales and males. Similarly, Ribas et al. reported that females exhibited more DEGs than males in heat-treated zebrafish [23]. Interestingly, differences among sexes are so obvious in only 48-h acute heat treatment. In *C. semilaevis*, ovarian differentiation can be observed earlier than the testicular differentiation. Histological observations show that gonadal differentiation begins at ~ 62 days post hatching (dph) [24]. At this stage, primordial germ cells (PGCs) begin mitosis rapidly to form clusters of oocytes. The appearance of the ovarian cavity is observed at ~ 100 dph. On the other hand, in deduced testis, PGCs start mitosis at ~ 80 dph to develop into spermatogonia, and testicular differentiation begins at ~ 100 dah [25]. In this study, 90 dph females showed more variable responses to heat stress, indicating that the early gonadal differentiation is vulnerable to high temperature, and different cell types may have different performance under heat stress.

The 1048 DEGs shared in females, pesudomales and males may represent the inherent difference between high and normal temperatures. Of these, several members of *hsp* gene family were found, which is consistent with previous studies [26, 27]. The Hsps, mainly Hsp70s and their chaperons, can re-fold thermally damaged proteins and prevent their cytotoxic aggregation [28, 29]. Potential regulators of *hsp* gene family were differentially expressed under high temperature. For example, RNA-seq showed that *eef1akmt3* was up-regulated in females and pseudomales, but down-regulated in males. The *eef1akmt4* was down-regulated in all three sexes, both in RNA-seq and qRT-PCR validation. The translation elongation factor, eEF1A participates in heat shock response (HSR) in mammalian cells. It can rapidly activate HSP70 transcription by recruiting HSF1 to its promoter, stabilize HSP70 mRNA and facilitate its transport from the nucleus to ribosomes [30]. The *eef1akmt3* and *eef1akmt4* are methyltransferase-related genes, which specifically target Lys-165 and Lys-36 in eEF1A [31, 32]. The differentially expressed *eef1akmt3* and *eef1akmt4* genes may indicate the change in the eEF1A methylation state and the eEF1A mRNA expression level.

In addition, some DEGs are sex-specific under heat stress. In transcriptome data of females, *kdm6b* was down-regulated after heat stress. *kdm6b* is an epigenetic regulator demethylating H3K27me3 at *dmrt1* promoter, which plays a causal role in male sex determination in red-eared slider turtle (*Trachemys scripta elegans*). The *kdm6b* can rapidly respond to temperature change, and it can be down-regulated in the high temperature [13]. In turtles, high temperature increased the production of females, while it increased the production of males in *C. semilaevis*. Our results may indicate *kdm6b* as a conserved responder to high temperatures among fish and reptiles. Its role in promoting masculinization of *C. semilaevis* under high temperatures deserves further study. Another candidate epigenetic regulator, *saml*, was specifically up-regulated in heat-treated pseudomales both in RNA-seq and qRT-PCR analyses. The S-adenosylmethionine synthase enzyme is sensitive to abiotic stress and catalyzes the S-adenosylmethionine synthesis, which provides methyl groups for the methylation of DNA, RNA and proteins [33]. These results may indicate the role of epigenetic regulation during the physiological response to high temperature.

Two cortisol related genes, *hsd11b2* and *hsd17b1* were specifically down-regulated in heat-treated females, both in RNA-seq and qRT-PCR analyses. Cortisol is a stress-related hormone, which plays an important role in high-temperature induced masculinization [9–11]. There is crosstalk between cortisol and androgen synthesis [34]. The *esrrα* and *ar* genes were down-regulated in heat-treated females, while *errγl* was specifically down-regulated in males. These changes in sex hormones indicate the role of cortisol and androgen pathways response to high temperature. However, expression of sex-related genes, such as *cyp19a1a*, *foxl2*, *dmrt1* and *amh* was not significantly different under heat stress. This may show that 48-h heat stress can only affect the upstream hierarchy but cannot change the expression level of sex-related genes.

## Conclusions

Our study revealed different performances among sexes under heat stress in *C. semilaevis*. Besides, several genes were identified to participate in high temperature perception by comparative transcriptome analysis, and some of them showed a sex-biased characteristic. These results may provide a clue for understanding the responses of genes and networks to environmental temperature and expand our current understanding of the effects of gonadal gene expression in teleosts. However, future studies are required to determine the role of these temperature-sensitive genes on sex determination and differentiation.

## Methods

### Heat stress treatment and sample collection

Three-month-old sex determined *C. semilaevis* were used in this study. The number of fish was determined in order to sample a sufficient number of pseudomales. The genetic female: male ratio was close to 1:1, and approximately 14% of genetic females could spontaneously sex revered into pseudomales [20, 21]. At least five pseudomales of both control and heat-stress groups with three biological replicates for each group were randomly selected for transcriptome sequencing. Thus, a total number of 140 fish were used in the experiment. Fish were obtained from Laizhou Mingbo Aquatic Co., Ltd., Yantai, China (body length: 5.457 ± 0.663 cm), transported to the laboratory and reared in 120 L tanks at 22 °C. After the acclimation period, fish were randomly divided into two groups. The heat stress group (HS, *n* = 70) was exposed to high temperature (28 °C) with a gradual increase of temperature (2 °C per hour) up to 48 h. The control group (CT, *n* = 70) was maintained at 22 °C. The temperature treatment used in this study would not induce pseudomale formation. After treatment, fish were anesthetized with 0.05% MS-222 (Sigma, Shanghai, China) via immersion bath and decapitation [35]. The gonad of each fish was dissected and immediately stored in liquid nitrogen for RNA extraction, and the caudal fin was stored in ethanol for genetic sex determination.

### Sex identification of *C. semilaevis*

The sex of each fish was identified after the heat treatment. Genomic DNA (gDNA) was extracted from the caudal fin using the phenol-chloroform method. The genetic sex was identified by PCR using sex-F and sex-R primers, which amplify two bands of 169 and 134 bp in females, and one band of 169 bp in males [22]. The pseudomales were distinguished from females by qRT-PCR against the sex-determining gene, *dmrt1* [36] (Additional file 4).

### Total RNA extraction, cDNA library construction and sequencing

Total RNA from each gonad was extracted using Trizol (Invitrogen, Carlsbad, USA) according to the manufacturer’s instructions. The quantity and quality of the RNA were determined using an Agilent 2100 bioanalyzer (Thermo Fisher Scientific, Santa Clara, USA). High-quality RNA (RIN > 7) was used for mRNA library construction via the conventional protocol. Three biological replicates for each group were used. The libraries of heat-stressed females (HS_F1–3), males (HS_M1–3) and pseudomales (HS_P1–3) were sequenced on BGISEQ-500 platform by PTM Biolabs (Hangzhou, China). The raw reads were deposited in NCBI’s Sequence Read Archive (SRA) database (BioProject no. PRJNA605682). The transcriptome data of CT group were reported in our previous study [37] and downloaded from SRA database (BioProject no. PRJNA576366).

### Quality control, mapping and annotation of sequencing reads

The quality of the raw reads was assessed using SOAPnuke v1.4.0 [38]. Adapters and low-quality bases were trimmed by Trimmomatic v0.36 [39]. All filtered clean reads from CT and HS groups were mapped to the *C. semilaevis* reference genome (NCBI, Cse_v1.0**,** BioProject no. PRJNA73987) using HISAT2 v2.1.0 [40] and aligned with the reference transcript sequence by Bowtie2 v2.2.5 [41].

### Identification of DEGs

Gene expression levels were estimated using RNA-Seq by Expectation Maximization, RSEM v1.2.8 [42], and Fragments Per Kilobase Million (FPKM) was calculated to represent the expression level of each gene. The DEGseq and DEseq2 were used to determine the DEGs [43, 44]. Genes with an adjusted *p*-value of less than 0.001 and fold change greater than 2 were defined as DEGs. PCA was performed using OmicShare tools (www.omicshare.com/tools). The heatmap of sex-biased genes was drawn by R Package pheatmap v1.0.12 (https://CRAN.R-project.org/package=pheatmap). The average expression level of three biological replicates was used to represent the corresponding group and conduct the PCA, DEG, GO enrichment and KEGG enrichment analyses.

### GO and KEGG pathway enrichment analyses

GO (http://www.geneontology.org/) and KEGG (http://www.kegg.jp/) enrichment analyses of annotated DEGs were performed by Phyper (http://en.wikipedia.org/wiki/Hypergeometric_distribution) based on Hypergeometric test. The threshold of *p* < 0.05 is considered to be significant.

### RNA-seq data validation by qRT-PCR

Nine candidate genes related to high-temperature response (*hspa1*, *hsf4*, *hsc70*, *eef1akmt4*, *saml*, *hsd11b2*, *hsd17b1*, *esrrα* and *ar*) were selected to validate the RNA-seq data by qRT-PCR. The primers were designed based on their sequences from the NCBI database (Additional file 5). *β-actin* gene was used as the internal control. One microgram of total RNA for high-throughput transcriptome sequencing was reverse transcribed into cDNA with the PrimeScript™ RT reagent Kit with gDNA Eraser (Takara, Japan). Then, qRT-PCR was performed using QuantiNova™ SYBR Green PCR Kit (Qiagen, Germany) in 20-μl reactions, containing 10 μl 2 × SYBR Green PCR Master Mix, 2 μl QN ROX Reference Dye, 0.7 μM forward primer, 0.7 μM reverse primer and 1 μl cDNA. The cycling program was carried out at 95 °C for 2 min, followed by 40 cycles of 95 °C for 5 s and 60 °C for 10 s; this was followed by a melting curve analysis in an ABI StepOnePlus Real-Time PCR system (Applied Biosystems, USA). Reactions were performed in triplicate. The relative expression fold changes of these genes were analyzed using the 2^−ΔΔCt^ method.

### Statistical analysis

The results of the qRT-PCR analysis were expressed as means ± S.E.M.. The values were compared by multiple *t*-test using GraphPad Prism 7.0 (GraphPad, USA). Statistically significant differences were defined as *p* < 0.05.

## Supplementary information

**Additional file 1.** Statistics of transcriptome sequencing. Data in females, pseudomales and males from each group (control group: CT_F, CT_P and CT_M; heat-stress group: HS_F, HS_P and HS_M) were listed.

**Additional file 2.** Shared and sex-specific genes involved in high temperature response. Interested differentially expressed genes (DEGs) shared in females, pseudomales and males, and DEGs specifically changed in females, pseudomales or males between control (CT) and heat-stress (HS) groups were listed.

**Additional file 3 **Enriched Gene Ontology (GO) terms and Kyoto Encyclopedia of Genes and Genomes (KEGG) pathways of sex-specific DEGs under heat stress. The differentially expressed genes (DEGs) specifically changed in females, pseudomales, or males in comparison of heat-stress (HS) vs. control (CT) groups were subjected to GO enrichment analysis and resulted significant GO terms (*p* < 0.05) were listed in Sheet 1. KEGG pathway enrichment analysis of these sex-specific DEGs was listed in Sheet 2 (*p* < 0.05).

**Additional file 4 **Sex identification of *C. semilaevis*. (A, B) Genetic sex identification of *C. semilaevis* using sex-linked SSR marker. (A) Genetic sex identification of *C. semilaevis* in the control (CT) group; (B) Genetic sex identification of *C. semilaevis* in the heat-stress (HS) group. Genetic females have two sex-linked SSR bands, while genetic males have only one band. (C, D) Relative expression level of male-determining gene *dmrt1* in females, pseudomales and males. (C) Relative expression level of *dmrt1* in CT group; (D) Relative expression level of *dmrt1* in HS group.

**Additional file 5.** The primers used in this study. The column of Gene ID lists the accession number of each gene in the NCBI database.

## Data Availability

The transcriptome data used in this study were uploaded to the National Center for Biotechnology Information (NCBI) Sequence Read Archive (SRA) with accession numbers PRJNA576366 and PRJNA605682. The high-quality *C. semilaevis* reference genome was downloaded from NCBI (Cse_v1.0, BioProject no. PRJNA73987). The sequences of genes selected for RNA-seq validating were acquired from the NCBI database with their corresponding GeneIDs [*hspa1* (103393458), *hsf4* (103379546), *hsc70* (103395053), *eef1akmt4* (103395991), *saml* (103387328), *hsd11b2* (103378825), *hsd17b1* (103393165), *esrrα* (103394741), *ar* (103395329), *β-actin* (103393304), *dmrt1* (103397807)].

## Abbreviations

CT: Control group
dah: Days after hatching
DEG: Differentially expressed gene
FPKM: Fragments per kilobase million
GO: Gene ontology
MS-222: Tricaine methanesulfonate
NCBI: National Center for Biotechnology Information
PCA: Principal component analysis
HS: Heat stress group
HSF1: Heat shock transcription factor 1
HSP: Heat shock protein
HSR: Heat shock response
KEGG: Kyoto Encyclopedia of Genes and Genomes
Lys: Lysine
PGCs: Primordial germ cells
qRT-PCR: Real-time quantitative-polymerase chain reaction
SRA: Sequence Read Archive
