# Supplementary material for: Early response to heat stress in Chinese tongue sole (Cynoglossus semilaevis): performance of different sexes, candidate genes and networks
Source: BMC Genomics. 2020 Oct 27;21:745. doi: 10.1186/s12864-020-07157-x (PMC7590793; doi:10.1186/s12864-020-07157-x)
Supplement: Supplementary file 1 — Additional file 1. Statistics of transcriptome sequencing. Data in females, pseudomales and males from each group (control group: CT_F, CT_P and CT_M; heat-stress group: HS_F, HS_P and HS_M) were listed. [file 12864_2020_7157_MOESM1_ESM.pdf]

Sample name	Total raw read (million)	Total clean read (million)	Clean read Q20 (%)	Clean read Q30 (%)	Clean read ratio (%)	Total mapping genome ratio (%)
CT_F1	73.27	67.22	97.72	89.90	91.73	88.58
CT _F2	73.46	67.36	97.66	89.64	91.69	88.66
CT _F3	77.04	69.94	97.10	88.25	90.79	89.25
CT _M1	74.60	68.36	97.73	89.94	91.64	88.28
CT _M2	75.82	69.43	97.63	89.57	91.57	89.50
CT _M3	73.23	66.73	97.62	89.52	91.12	89.02
CT _P1	75.91	69.52	97.65	89.62	91.58	88.25
CT _P2	75.93	69.24	97.52	89.13	91.20	88.19
CT _P3	77.13	70.05	97.14	88.42	90.82	88.63
HS_F1	74.71	66.77	96.86	88.05	89.37	89.34
HS_F2	72.99	64.86	96.66	87.37	88.86	89.52
HS_F3	77.20	69.13	96.92	88.20	89.55	89.04
HS_M1	74.64	67.71	97.00	87.98	90.71	89.15
HS_M2	74.64	67.73	97.03	88.03	90.74	89.07
HS_M3	77.00	69.45	96.94	87.78	90.20	89.20
HS_P1	74.71	66.84	96.78	87.78	89.46	88.64
HS_P2	77.20	69.17	96.84	87.92	89.60	89.12
HS_P3	74.71	66.73	96.84	87.91	89.31	88.15
