# Supplementary figures and images for: Early response to heat stress in Chinese tongue sole (Cynoglossus semilaevis): performance of different sexes, candidate genes and networks
Source: BMC Genomics. 2020 Oct 27;21:745. doi: 10.1186/s12864-020-07157-x (PMC7590793; doi:10.1186/s12864-020-07157-x)

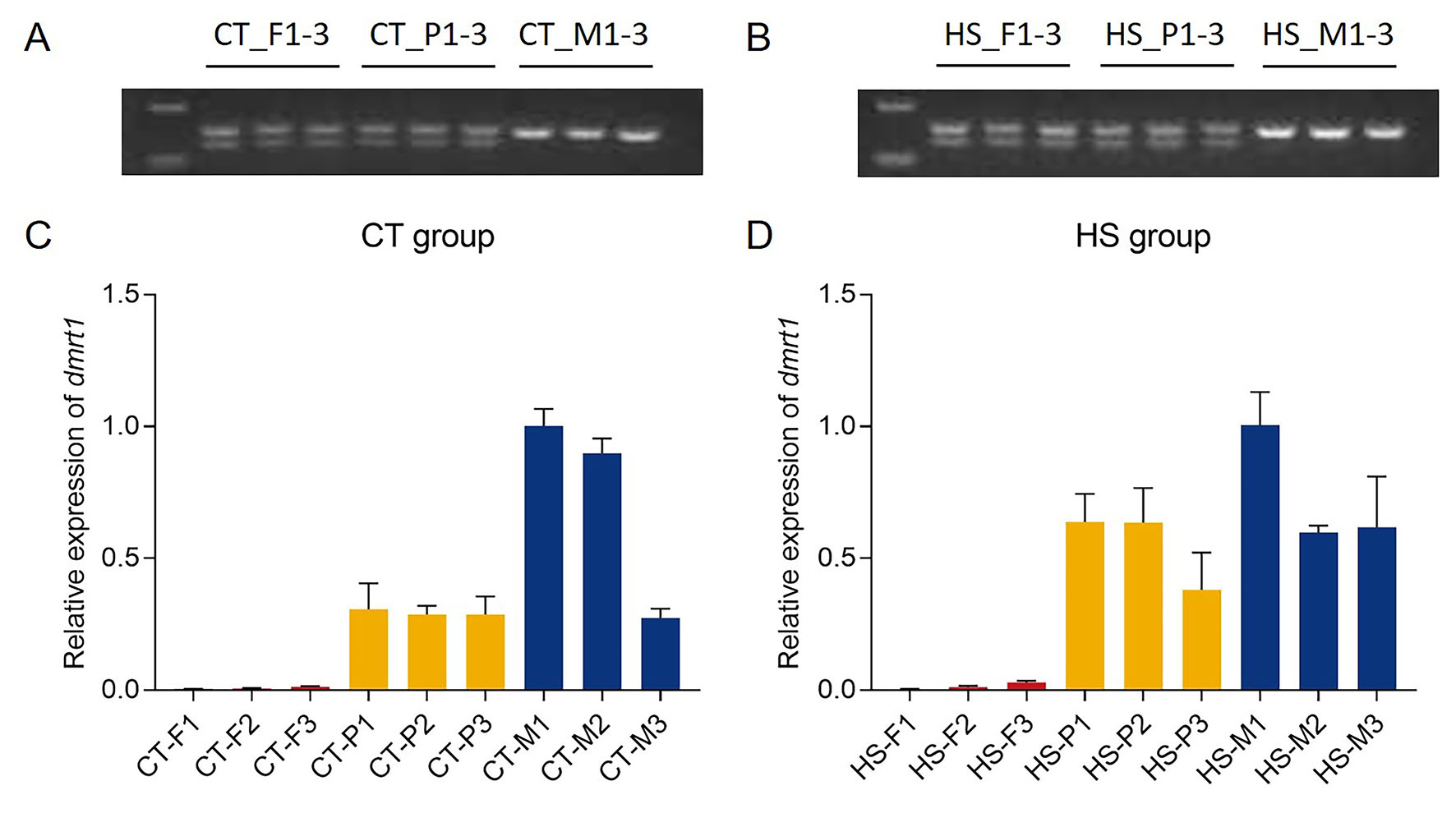

Supplement: Supplementary file 4 — Additional file 4 Sex identification of C. semilaevis. (A, B) Genetic sex identification of C. semilaevis using sex-linked SSR marker. (A) Genetic sex identification of C. semilaevis in the control (CT) group; (B) Genetic sex identification of C. semilaevis in the heat-stress (HS) group. Genetic females have two sex-linked SSR bands, while genetic males have only one band. (C, D) Relative expression level of male-determining gene dmrt1 in females, pseudomales and males. (C) Relative expression level of dmrt1 in CT group; (D) Relative expression level of dmrt1 in HS group. [file 12864_2020_7157_MOESM4_ESM.jpg]
