# Supplementary material for: Early response to heat stress in Chinese tongue sole (Cynoglossus semilaevis): performance of different sexes, candidate genes and networks
Source: BMC Genomics. 2020 Oct 27;21:745. doi: 10.1186/s12864-020-07157-x (PMC7590793; doi:10.1186/s12864-020-07157-x)
Supplement: Supplementary file 5 — Additional file 5. The primers used in this study. The column of Gene ID lists the accession number of each gene in the NCBI database. [file 12864_2020_7157_MOESM5_ESM.pdf]

Primer name	Primer sequence (5'-3')	Usage	Gene ID
hspa1-qF	GGAACTCTGGAACCTGTGGA	qRT validation	103393458
hspa1-qR	ATGCTCTTGTTCAGCTCCCT		
hsf4-qF	GGTTAAGAGCGAGGAGACCA	qRT validation	103379546
hsf4-qR	CAGTGAGACCACCTCTCTCC		
hsc70-qF	CTGCTGCTTCTGGATGTCAC	qRT validation	103395053
hsc70-qR	GGCTGGTTGTCTGAGTAGGT		
eef1akmt4-qF	ATTGGTTGGGCACTTTGTCC	qRT validation	103395991
eef1akmt4-qR	TGGCTGCCATCGTGTTTATG		
hsd11b2-qF	TCCATCATCCTGCCCTCATC	qRT validation	103378825
hsd11b2-qR	TGAGGTCTGGGTTAGCTTGG		
hsd17b1-qF	CTAGTGTCGGTGTGCATGTG	qRT validation	103393165
hsd17b1-qR	CCCAGCTGTAACACAGGAGA		
saml-qF	ACCAGAGTTCACCTGGGAAG	qRT validation	103387328
saml-qR	TTACAGGCAGTGTTGTGTGC		
esrra-qF	TAGTGTGTGGAGACGTTGCT	qRT validation	103394741
esrra-qR	TGGTAAAGCGACATGCTTGG		
ar-qF	AGGTTCGAAACAGGCAGAGA	qRT validation	103395329
ar-qR	GTTTGCCTTCTGCAGCTCTT		
β-actin-qF	GCTGTGCTGTCCCTGTA	qRT validation	103393304
β-actin-qR	GAGTAGCCACGCTCTGTC		
dmrt1-qF	CCGGACGGCTTCGTGTC	Sex identification	103397807
dmrt1-qR	CTTCCACAGGGAGCAGGCAGT		
Sex-F	CCTAAATGATGGATGTAGATTCTGTC	Sex identification	/
Sex-R	GATCCAGAGAAAATAAACCCAGG		

Note: The column of Gene ID lists the accession number of each gene in the NCBI database.
